# Copper-enriched zinc peroxides induced cuproptosis through concurrent metabolic and oxidative dysregulation for boosting immunotherapy in colorectal cancer

**DOI:** 10.1016/j.mtbio.2026.102830

**Published:** 2026-01-19

**Authors:** Shaopeng Zhang, Shaokang Yang, Mingqi Li, Hao Zhang, Yue Cao, Shiqi Bai, Wei Li, Bin Wang, Donghao Qu, Ziqian Wang, Wanying Li, Yanxu Sun, Daguang Wang, Yinghui Wang, Hongjie Zhang

**Affiliations:** a Department of Gastrocolorectal Surgery, General Surgery Center, The First Hospital of Jilin University, Changchun 130021, China; b Department of Colorectal Surgery, Harbin Medical University Cancer Hospital, No.150 Haping Road, Harbin, Heilongjiang, 150081, China; c Department of Central Laboratory, The Affiliated Huaian No.1 People's Hospital Nanjing Medical, Huai'an, 223300, Jiangsu Province, China; d Department of Neurosurgery, The First Hospital of Jilin University, Changchun 130021, China; e Genetic Diagnosis Center, The First Hospital of Jilin University, Changchun 130021, China; f State Key Laboratory of Rare Earth Resource Utilization, Changchun Institute of Applied Chemistry (CIAC), Chinese Academy of Sciences, Changchun 130022, China; g Operating Room Surgery, Harbin Medical University Cancer Hospital, No.150 Haping Road, Harbin, Heilongjiang, 150081, China

**Keywords:** Colorectal cancer, Cuproptosis, Glycolysis, Immunotherapy, Nanomedicine

## Abstract

Despite the immunotherapy has achieved the progress for advanced colorectal cancer, the unsatisfactory treatment effect remains a challenge due to the deficient immune response. In this work, we constructed a tumor microenvironments (TME)-responsive biodegradable cuproptosis inducer (ZnO_2_-Cu@HA, ZCH) through cation-exchange method for amplifying the immune response. Compared to free copper ions, ZCH cloud achieve the controllable release of Cu^2+^ in tumor site, trggering efficient cuproptosis but reducing the side effect of normal tissues. Furthermore, the released Zn^2+^ could also inhibit intracellular glycolysis and ATP generation, then block the ATP7B to reduce the efflux of copper ions. Meanwhile, ZCH broke intracellular redox homeostasis via the release of exogenous H_2_O_2_, Cu^+^-mediated Fenton-like reaction and Zn^2+^-induced endogenous mitoROS, amplifying the cuproptosis to inducing immunogenic cell death (ICD) triggered for highly efficient immunotherapy of colorectal cancer. These findings demonstrated that it is a promising strategy of inducing efficient cuproptosis by the synergistic effect of accumulation of copper ions, inhibiting glycolysis and down-regulation GSH for efficient immunotherapy of colorectal cancer.

## Graphical abstract

A novel TME-responsive biodegradable nanoplatform (ZnO_2_-Cu@HA, ZCH) was constructed by doping copper ions through cation-exchange method before functionalized with hyaluronic acid (HA) for inducing the efficient antitumor immune response mediated by cuproptosis. The nanoplatform inducing cuproptosis by accumulation of copper ions, inhibiting glycolysis and down-regulation GSH has a promising potential to induce immune response for boosting colorectal cancer immunotherapy.

**Figure:**
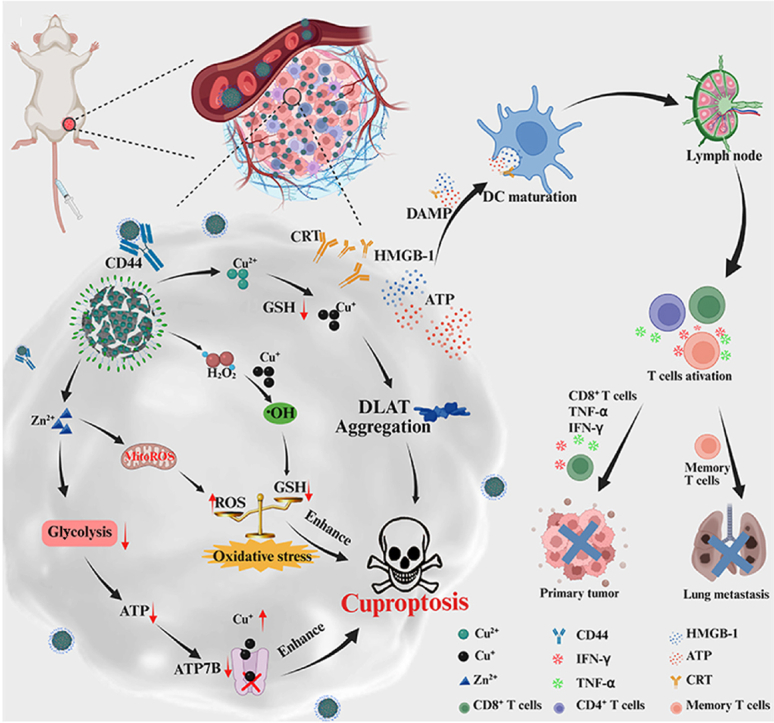

## 1 Introduction

Colorectal cancer is the third most prevalent malignant gastrointestinal tumor, posing a serious threat to the survival of patients [[1], [2], [3]]. In the last decades, immunotherapy for colorectal cancer has made tremendous progress, providing a promising approach for the treatment of colorectal cancer [4,5]. However, the limited antitumor immune response resulted in the unsatisfactory efficacy of immunotherapy. It is crucial to develop the new strategy for provoking highly efficient antitumor immune response. Cuproptosis, a novel non-apoptotic program cell death, leads to the aggregation of lipoylated proteins and the loss of Fe-S cluster proteins through binding copper ions with lipoylated proteins of the tricarboxylic acid (TCA) cycle, further inducing the proteotoxic stress and cell death [6]. The recent researches had demonstrated that cuproptosis can activate an adaptive immune response by induction of immunogenic cell death (ICD), further enhancing systemic anticancer immunity [[7], [8], [9], [10]]. The elesclomol, one of copper ionophores, could transport amounts of copper ions into cells and trigger cuproptosis, exhibiting the potential in cancer treatment [11,12]. However, elesclomol, as a small molecule drug, suffers from the rapid clearance and nonspecific delivery, resulting in the limited treatment mediated by cuproptosis [13]. Copper based nanoparticles, such as Cu_2_O [14,15], CuO_2_ [16,17] and copper-based metal organic framework (MOF) [18], could release copper ions in response to TME, triggering cuproptosis to kill the tumor cells. Therefore, it is a promising strategy to construct the copper-based nanoparticles with the capability of releasing Cu^2+^ in responsive to TME for inducing efficient cuproptosis. However, the cancer cells are insensitive to cuproptosis compared to normal cells, due to the Warburg's effect that cancer cells obtain sufficient supply of energy through aerobic glycolysis rather than oxidative phosphorylation respiration [19]. Since cuproptosis efficacy is dependent on the mitochondrial oxidative phosphorylation level [20], the glycolysis inhibition could also enhance cuproptosis [21]. In addition, glycolysis inhibition could block the ATP synthesis and down-regulate Cu^+^-transporting ATP7B, one of ATP-dependent transmembranous pumps, further resulting in the reduced intracellular copper efflux [[22], [23], [24]]. Moreover, glutathione (GSH), an antioxidant overexpressed in tumor cells [25], could significantly restrict the effect of cuproptosis [26,27]. Therefore, it is highly desired to developed the intelligent nanoplateform with multifunctions of Cu^2+^ delivery, glycolysis inhibition as well as down-regulation GSH for highly effective induction of cuproptosis.

Recent studies demonstrated that high level of Zn^2+^ in tumor cells can achieve effective glycolysis inhibition by impairing the functioning of lactate dehydrogenase (LDH) and glyceraldehyde-3-phosphate dehydrogenaseic enzymes (GAPDH) [[28], [29], [30], [31]]. Meanwhile, Zn^2+^ also can induce the generation of ROS by inhibiting the electron transport chain of mitochondria [32,33], thereby breaking the intracellular redox homeostasis. However, the limited ROS production by Zn^2+^ is difficult to disrupt cuproptosis defense. Moreover, the ROS generation ability of Cu^2+^ remains subject to the level of H_2_O_2_ in TME. The zinc peroxide nanoparticles (ZnO_2_ NPs), one of metal peroxides, can be degraded in acidic TME to release Zn^2+^ and hydrogen (H_2_O_2_), which can cause the generation of ROS and depletion of GSH [34]. In addition, the ZnO_2_ NPs was successfully doped with metal ion including Mn^2+^ and Fe^3+^ via a cation-exchange method, achieving TME-responsive H_2_O_2_ self-supply and metal ions release, enhancing ROS generation to disrupt the redox homeostasis [[35], [36], [37]]. Recent advances in Zn/Cu bimetallic nanomaterials have shown promise for antitumor immunotherapy, but none have exploited Zn^2+^-mediated metabolic reprogramming as a synergistic mechanism to enhance anti-tumor outcomes [38,39]. Therefore, constructing an intelligent and biodegradable nanoplatform based on Cu^2+^ doped ZnO_2_ NPs would achieve the precise delivery of Cu^2+^, regulation of redox homeostasis, and glycolysis inhibition for highly efficient colorectal cancer immunotherapy via inducing cuproptosis. However, there is no report on the cuproptosis induced based on Cu^2+^ doped ZnO_2_ NPs for efficent immunotherapy of colorectal cancer till now as we known.

**Scheme 1:**
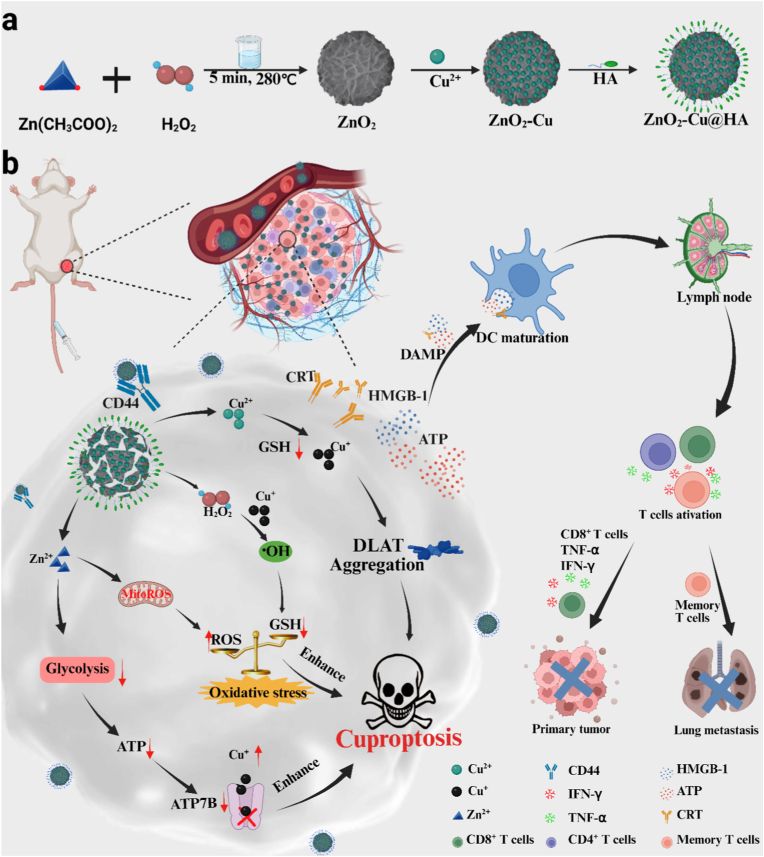
(a) Schematic illustration for the Synthesis of ZCH. (b) schematic illustration of multifunctional nanoplatform for eliciting antitumor immune response by inducing cuproptosis through accumulation of copper ions, inhibiting glycolysis and breaking intracellular redox homeostasis.

Herein, we constructed a TME-responsive biodegradable nanoplatform (ZnO_2_-Cu@HA, ZCH) by doping copper ions through cation-exchange method before functionalized with hyaluronic acid (HA) for inducing the efficient antitumor immune response mediated by cuproptosis (Scheme 1). After intravenous injection, ZCH could efficiently accumulate at the tumor site by targeting CD44 overexpressed in colorectal cancer and enhanced permeability and retention (EPR) effect [[40], [41], [42], [43]]. In TME, ZCH degraded in response to the weak acidic conditions, resulting in the release of H_2_O_2_, Cu^2+^ and Zn^2+^. Cu^2+^ was reduced to more toxic Cu^+^ by GSH, which not only induce cuproptosis, but also convert H_2_O_2_ to hydroxyl radicals (•OH) through Fenton-like reaction. Then, accumulation of ROS break intracellular redox homeostasis and deplete the intracellular GSH, which could be enhanced by Zn^2+^-mediated endogenous ROS production in mitochondria and released H_2_O_2_. In addition, Zn^2+^ could effectively impair the functioning of LDH and GAPDH, inhibit glycolysis and production of intracellular ATP, resulting in the following blocking of ATP7B. The combination of regulating redox homeostasis and inhibiting glycolysis effects could significantly amplify cuproptosis, and further induce ICD for achieving the highly efficient immunotherapy of colorectal cancer. Above all, this work provides a new avenue to amplifying cuproptosis by the synergistic effect of regulating redox homeostasis and inhibiting glycolysis for efficient immunotherapy of colorectal cancer.

## 2 Result and discussion

### 2.1 Synthesis and characterizations

**Fig. 1:**
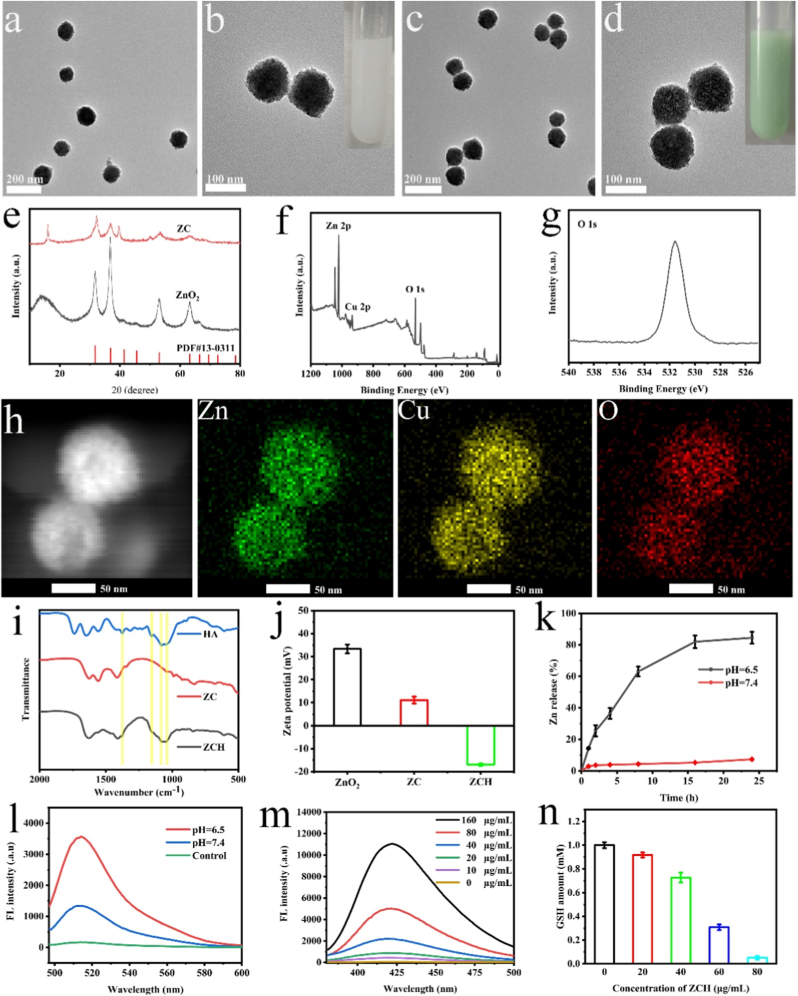
Characterization of ZCH. Representative TEM images of ZnO_2_ NPs (a) (b) and ZCH NPs (c) (d). (e) XRD patterns of ZnO_2_ NPs and ZC NPs. (f) Full XPS spectrum of ZC NPs. (g) O 1s spectrum of ZC NPs (h) The elemental mapping of ZC NPs. (i) The FT-IR spectra of ZC NPs, HA NPs and ZCH NPs. (j) The Zeta potentials of ZnO_2_ NPs, ZC NPs and ZCH NPs. (k) The release behavior of Zn element from ZCH NPs under different conditions. (l) Fluorescence spectra representing H_2_O_2_ production of ZCH NPs under different conditions. (m) Hydroxyl radical generation of different concentrations ZCH NPs. (n) GSH depletion capacity of different concentrations ZCH NPs.

ZnO_2_ NPs with an average size of 100 nm were synthesized by method of underwater Leidenfrost dynamic chemistry (Fig. 1a and b) [44]. Then, the Cu-doped ZnO_2_ NPs (ZC) was construct through cation-exchange approach, which were functionalized with HA to synthesize ZnO_2_-Cu @HA (ZCH)**.** As shown in Fig. 1c and d, ZCH was uniformly dispersed and showed negligible change in morphology and size compared to ZnO_2_ NPs. As shown in the X-ray diffraction (XRD) patterns (Fig. 1e), the crystal structure of ZnO_2_ completely matched diffraction peaks to ZnO_2_ (PDF#13–0311). Meanwhile, the pattern of ZC reveals that the enrichment of Cu^2+^ has no obvious impact on the crystal structure of ZnO_2_. The result from X-ray photoelectron spectroscopy (XPS) further confirmed the above results (Fig. 1f, g, S1 and S2). The spectrum of O 1s peak displayed the characteristic binding energy peaks at 531.9 eV, which can be recognized to O_2_^2−^ (Fig. 1g). The remarkable peaks at 933.8 eV and 953.6 eV were consistent with the binding energies of Cu (II)2p3/2 and Cu (II)2p1/2, respectively (Fig. S1). The elemental mappings of ZC demonstrated the existence of Cu, Zn, and O elements, further conforming the successful incorporation of Cu^2+^ (Fig. 1h). The ratio of Zn (366.3 ± 11.4 μg/mL) and Cu (170.7 ± 8.5 μg/mL) in ZCH (1000 μg/mL) was calculated to be approximately 2:1 by the inductively coupled plasma-optical emission spectroscopy (ICP-OES, Table S1). In order to improve the target ability and biocompatibility, HA was successfully functionalized on the surface of ZC, which was confirmed by the results of zeta potentials and fourier transform infrared spectroscopy (FT-IR) (Fig. 1i and j). The average hydrodynamic size of ZCH was 102 nm, 105 nm and 106 nm in water, PBS and RPMI 1640 medium, respectively, which would favor their tumor passive targeting and enrichment (Fig. S3). In addition, the mean diameter size did not change significantly after being dispersing in H_2_O, PBS and cell culture medium (RPMI 1640) for 7 days, indicating that ZCH has good stability under physiological conditions (Fig. S4). Therefore, all these results verified the successful construction of ZCH.

In order to investigate the stability, ZCH was incubated in the solutions with pH 6.5 and 7.4, respectively. As shown in Fig. S5, the structure of ZCH remained intact in neutral condition, but it collapsed completely under acidic conditions, which indicated its good degradation ability in TME. Additionally, the ion release in response to TME was further conducted by ICP-OES. Nearly 80 % of Zn^2+^ and Cu^2+^ were released from the ZCH in the buffer (pH 6.5), which is significantly higher than that in the buffer (pH 7.4). This result demonstrated its ability of controllable drug release in tumor site (Fig. 1k and S6). Similarly, the more H_2_O_2_ was detected in pH 6.5 buffer after incubation for 12 h, which could provide sufficient substrate for generating •OH (Fig. 1l). Then, terephthalic acid (TA) was employed to detect •OH generation upon incubation of ZCH in PBS (pH = 6.5) containing GSH (2 mM). The generation of •OH enhanced with the increasing of ZCH concentration (Fig. 1m). Next, we evaluated the GSH consumption ability of ZCH by GSH Assay Kit. With the increasing concentration of ZCH, the level of GSH gradually decreased, indicating the concentration-dependent GSH depletion capacity of ZCH (Fig. 1n). In brief, ZCH decomposed to Zn^2+^, Cu^2+^ and H_2_O_2_ in response to TEM, further producing •OH and depleting GSH.

### 2.2 Antitumor efficacy and mechanism in vitro

**Fig. 2:**
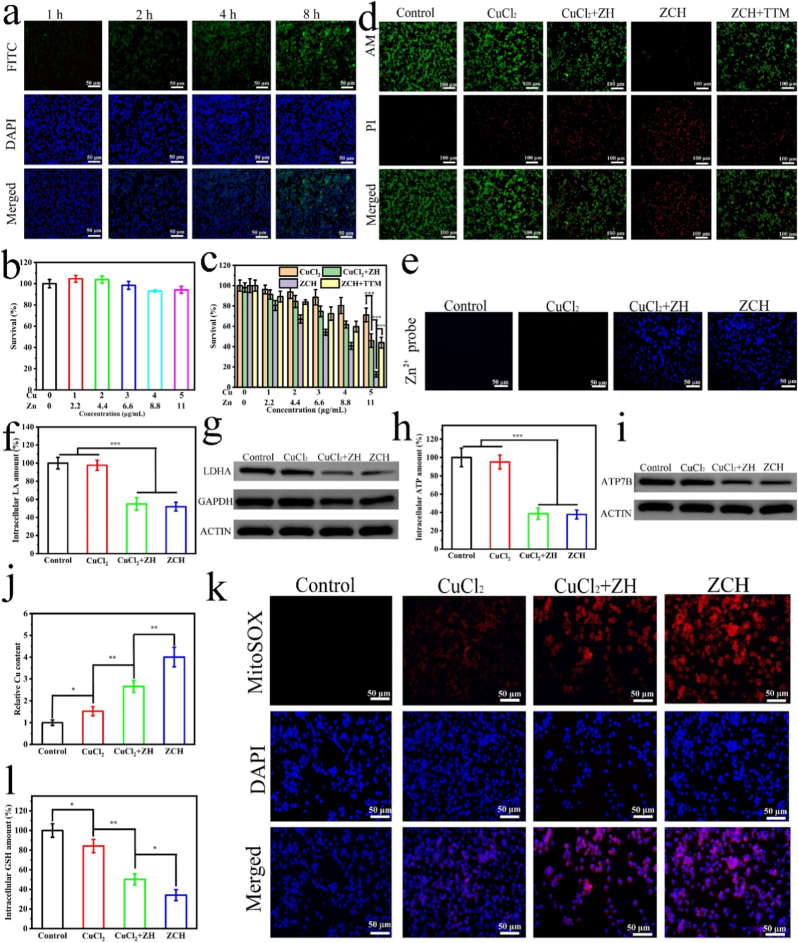
ZCH NPs inhibited glycolysis and broke intracellular redox homeostasis (a) Fluorescent microscope images of cellular internalization of ZCH after incubating in CT26 for different times (Scale bar: 50 μm). (b) The cytotoxicity of ZCH NPs toward L929 cells after incubating for 24 h. (c) The therapeutic effect of different nanomaterials with different concentrations. (d) Fluorescent microscope images of CT26 cells co-stained with Calcein AM and PI after different treatments (Scale bar: 100 μm). (e) The detection of intracellular Zn^2+^ in CT26 cells after treated with different nanomaterials (Scale bars: 50 μm). (f) The detection of intracellular LA content of CT26 cells in different groups. (g) Western blotting analysis of the GAPDH and LDHA expression in CT26 cells treated with different nanomaterials. (h) The detection of intracellular ATP content of CT26 cells in different groups. (i) Western blotting analysis of the ATP7B expression in CT26 cells treated with different nanomaterials. (j) The detection of intracellular Cu content of CT26 cells in different groups. (k) The mitoROS generated in CT26 cells treated with different nanomaterials (Scale bars: 50 μm). (l) The detection of intracellular GSH content of CT26 cells in different groups.∗p < 0.05, ∗∗p < 0.01, and ∗∗∗p < 0.001.

Encouraged by excellent performances of ZCH, we further investigated its antitumor ability in vitro. Initially, fluorescent probe fluorescein isothiocyanate (FITC) labeled ZCH was applied to explore the cellular uptake behaviors of ZCH in CT26 tumor cells. The intensity of intracellular green fluorescence signal increased with the incubation time (Fig. 2a and Fig. S7), which was critical for achieving subsequent anti-tumor therapy. The cell counting kit 8 (CCK-8) assay was performed to evaluate cytotoxicity and therapeutic effect of ZCH in vitro. ZCH showed negligible cytotoxicity to mouse fibroblast cells (L929) after incubation for 24 h, revealing the good biocompatibility of ZCH (Fig. 2b). Furthermore, the CT26 cells were used to test the therapeutic effect of ZCH. As shown in Fig. 2c, only 8 % of cells remained surviving in the ZCH group, which was lower than 40 % in the CuCl_2_+ZH group and 77 % in the CuCl_2_ group. The tetrathiomolybdate (TTM), as a copper chelator, attenuated the in vitro antitumor efficacy of ZCH. The excellent anti-tumor effect of ZCH may be ascribed to the better uptake of ZCH by CT26 cells than free Cu^2+^. Moreover, the result of live-dead cell staining and migration assay further demonstrated the largest lethality in ZCH group, which was also consistent with above results (Fig. 2d and Fig. S8).

In order to explore the antitumor mechanisms of ZCH, we firstly detected the level of Zn^2+^ in cells by Zinquin ethyl ester (a specific Zn^2+^ indicator). Conspicuously, compared to the control and CuCl_2_ group, the significant blue fluorescence was observed in CuCl_2_+ZH and ZCH group, indicating that ZCH and ZH could degrade to release of Zn^2+^ in CT26 cells (Fig. 2e and Fig. S9). Then we further determined the level of intracellular LA to assess the inhibition of glycolysis of ZCH. As expected, compared to control and CuCl_2_ group, a distinct decrease of LA levels was detected in CuCl_2_+ZH and ZCH group, illustrating the block of glycolysis mediated by Zn^2+^ (Fig. 2f and Fig. S10). Meanwhile, the western blot assay showed the up-regulation of GAPDH and LDHA, further demonstrating the inhibition of glycolysis and leading to the increase sensitivity of cancer cells to cuproptosis [[22], [23], [24]] (Fig. 2g). Since cancer cells obtain sufficient supply of energy through aerobic glycolysis rather than oxidative phosphorylation respiration [19], we further investigated the intracellular ATP level in CT26 cells after different treatments. As expected, the intracellular ATP level in CuCl_2_+ZH and ZCH group was significantly restrained (Fig. 2h), further down-regulated the expressions of ATP7B (Fig. 2i). Given that the high intracellular level of copper is indispensable for cuproptosis, ICP-OES was used to detect the intracellular copper contents after various treatments for 12 h. As shown in Fig. 2j, the level of copper in CuCl_2_+ZH group was higher than that in CuCl_2_ group, which could be ascribed the reduction of copper ion efflux mediated by down-regulating ATP7B expression. Notably, the most amount of copper was detected in ZCH group, which was attributed to the better uptake of ZCH by tumor cells compared to free copper ions. These results implied that ZCH could inhibit glycolysis, block ATP synthesis, down-regulate ATP7B expression and lead to intracellular excessive accumulation of copper ions.

It was worth mentioning that the generation of ROS could break intracellular redox homeostasis, reduce the level of endogenous GSH, further increase sensitive to cuproptosis [[45], [46], [47]]. First, 2′,7′-dichlorofluorescin diacetate (DCFH-DA) was used as a probe to detect the intracellular ROS generation [48]. As shown in Figs. S11 and S12, CT26 cells incubated with CuCl_2_ displayed weak green fluorescence compared with control cells, which could be caused by Fenton-like reaction of copper ions. Moreover, since exogenous H_2_O_2_ provided the substrate for the Fenton-like reaction, and Zn^2+^ increased mitochondrial ROS (mitoROS) production by inhibiting the electron transport chain of mitochondria [32,33], the stronger green fluorescence was observed in CuCl_2_+ZH group. Interestingly, CT26 cells incubated with ZCH displayed the greatest green fluorescence compared with other groups, which could be attributed to the excellent uptake of ZCH by tumor cells, releasing more Cu^2+^, Zn^2+^ and H_2_O_2_ for production ROS. Subsequently, we detected the mitoROS production using MitoSOX as a mitochondrial-specific ROS indicator according to the previous methods [29]. As expected, apparent red fluorescence displayed in CuCl_2_+ZH and ZCH group, indicating that the released Zn^2+^ induced the generation of mitoROS (Fig. 2k and Fig. S13). Interestingly, weak red fluorescence of mitoROS was seen in CuCl_2_ group, which could be attributed to the fact that intracellular ROS produced by Fenton-like reaction of copper ions caused a certain degree of mitochondrial stress [49]. Given the fact that the consumption of GSH was a significant factor for inducing cuproptosis [45], we further detected the intracellular GSH depletion by assay kit. As shown in Fig. 2l, the GSH depletion in the ZCH group reached 66 %, which is higher than CuCl_2_+ZH group and CuCl_2_ group. This phenomenon further proved ZCH with the good capacity of dynamically breaking intracellular redox homeostasi and down-regulation intracellular GSH.

**Fig. 3:**
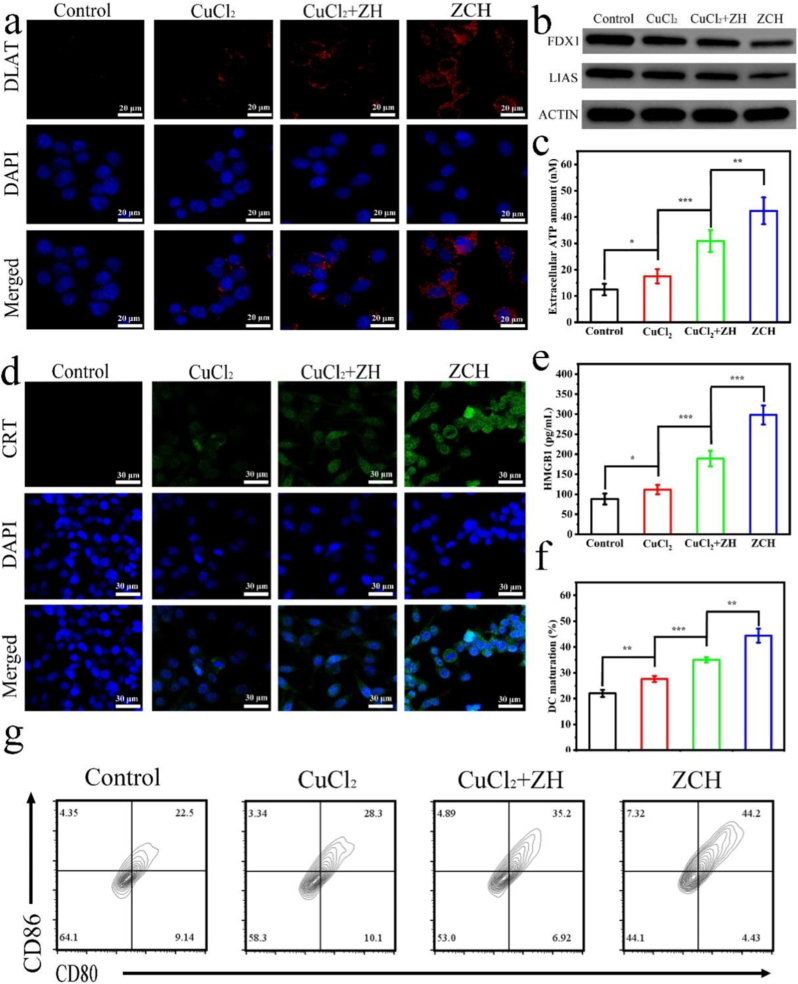
ZCH NPs induced cuproptosis and ICD. (a) Immunofluorescence images of intracellular DLAT with different nanomaterials (Scale bars: 20 μm). (b) Western blotting analysis of the FDX1 and LIAS expression in CT26 cells treated with different nanomaterials. (c) The release of ATP from CT26 cells after treated with different nanomaterials. (d) The detection of CRT exposure in CT26 cells after treated with different nanomaterials (Scale bars: 20 μm). (e) The release of HMGB1 from CT26 cells after treated with different nanomaterials. (f) the quantitative analysis (g) Flow cytometry analysis of DCs maturation in different groups.Intracellular. ∗p < 0.05, ∗∗p < 0.01, and ∗∗∗p < 0.001.

In order to demonstrate the cuproptosis induced by ZCH in CT26 cells, we studied the lipoylated dihydrolipoamide S-acetyltransferase (DLAT) oligomerization and destabilization of Fe-S cluster proteins [8,45]. The immunofluorescence imaging was performed to visualize DLAT oligomerization. As expected, the ZCH group exhibited the brightest green fluorescence, indicating the most amount of oligomerization of DLAT (Fig. 3a and Fig. S14). Furthermore, the result of western blotting demonstrated that ferredoxin (FDX1) and lipoyl synthase (LIAS) was least in ZCH group compared with other groups (Fig. 3b). The reduce of Fe-S cluster protein and the oligomerization of DLAT protein proved the occurrence of cuproptosis in CT26 cells. Given the fact that cuproptosis can induce ICD for activating an adaptive immune response [[7], [8], [9], [10]], we investigated the damage-related molecular pattern (DAMP) release, such as calreticulin (CRT), adenosine triphosphate (ATP), and high mobility group protein B1 (HMGB-1) in CT26 cells treated by different nanomaterials. As shown in Fig. 3c–e and Fig. S15, ZCH group released to the most DAMPs, indicated that cuproptosis mediated by ZCH could trigger ICD and evoke the subsequent immune response for antitumor treatment. Then we investigated the maturity of DCs in vitro by co-incubating bone-marrow-derived DCs (BMDCs) with the supernatant of CT26 cells treated by different treatments. As shown in Fig. 3f and g, the DCs maturation rate was 50.2 % in ZCH group, higher than CuCl_2_+ZH and CuCl_2_ group, revealing that the ICD induced by cuproptosis could efficiently promote DCs maturation. Therefore, all these above findings manifested that ZCH could activate efficient cuproptosis by accumulation of copper ions, inhibiting glycolysis and breaking intracellular redox homeostasis, further inducing the ICD of CT26 cells.

### 2.3 Antitumor efficacy in vivo

**Fig. 4:**
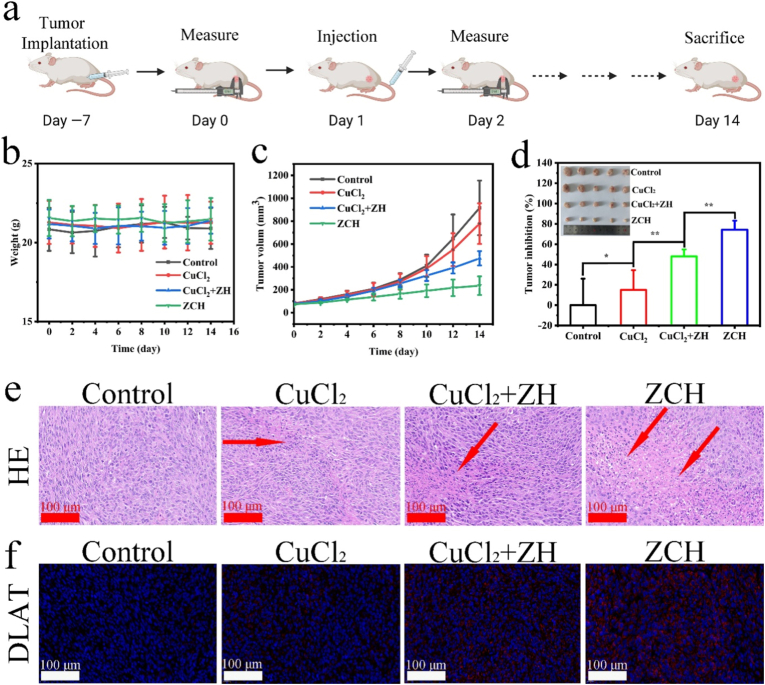
Antitumor effect of ZCH in vivo. (a) Schematic illustration of therapeutic protocol and monitor cycle. (b) Body weight curves of mice after different treatments. (c) Tumor volume curves and (d) tumor inhibition of mice after different treatments (Insert Image: the tumors from mice after different treatments). (e) H&E and (f) DLAT staining of tumor tissue after different treatments (Scale bar: 100 μm). ∗p < 0.05, ∗∗p < 0.01, and ∗∗∗p < 0.001.

Encouraged by the outstanding anti-tumor efficacy and immune activation in vitro, we further evaluated the therapeutic effect of ZCH in CT26 mice model (Fig. 4a). The female BALB/c mice bearing CT26 tumor were injected with different nanomaterials through the tail vein: (1) PBS solution, (2) CuCl_2_, (3) CuCl_2_+ZH and (4) ZCH. There is no obvious change in the weight of the mice, demonstrating the negligible toxicity on mice (Fig. 4b). Compared to the other groups, the ZCH group displayed the best the inhibition effect of tumor growth, which could be attributed the synergistic activating cuproptosis by accumulation of copper ions, inhibiting glycolysis and breaking intracellular redox homeostasis (Fig. 4c, d and Fig. S16). In addition, the most serious damage was found in hematoxylin and eosin (H&E) staining of tumor sections from ZCH group (Fig. 4e). Then, we evaluated the expression of DALT in tumor tissues by immunofluorescence staining. As shown in Fig. 4f, the brightest red fluorescence was seen in the tumor tissue sections of ZCH group, demonstrating the efficient cuproptosis mediated by ZCH in vivo. There was no significant difference in H&E staining of major organs between the different groups of mice, indicating the negligible toxicity and excellent biocompatibility of ZCH (Fig. S17). This result was further proven by the blood biochemistry assay of mice injected intravenously with ZCH for 30 days (Fig. S18). Therefore, these results validated the excellent antitumor effect of ZCH in vivo with low long-term toxicity.

### 2.4 Immune response induced by ICD in vivo

**Fig. 5:**
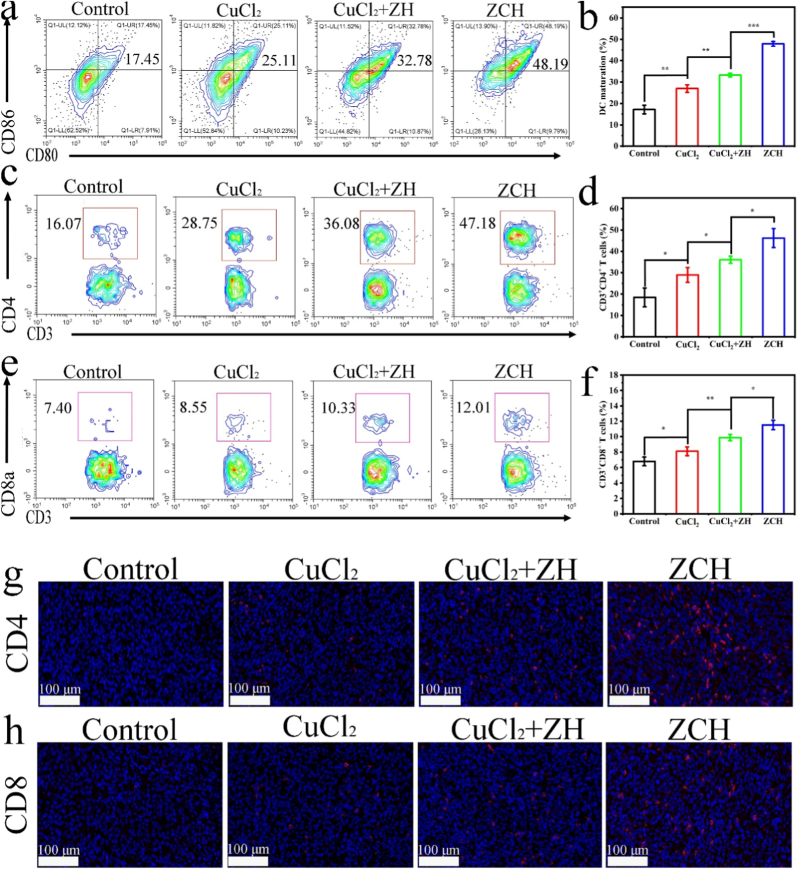
The immune response triggered by cuproptosis in vivo. (a) Flow cytometry analysis and (b) quantification of mature DCs in TDLNs from different groups. (c) Flow cytometry analysis and (d) quantification of CD4^+^ T cells in tumor from different groups. (e) Flow cytometry analysis and (f) quantification of CD8^+^ T cells in tumor from different groups. (g) Immunofluorescence staining of CD8^+^ and CD4^+^ cells in the tumor tissue from different groups (Scale bar: 100 μm). ∗p < 0.05, ∗∗p < 0.01, and ∗∗∗p < 0.001.

In order to investigate the immune response induced by ICD in vivo, the lymph nodes and tumor were obtained for next analysis. Considering the fact that ZCH can successfully activate cuproptosis to induce the ICD for promoting DCs maturation in vitro, the immunofluorescence staining was performed to detect the release of HMGB1 and the exposure CRT at tumor sites (Figs. S19 and S20). As expected, the ZCH treatment induced the most HMGB1 and CRT release among all groups, which could be attributed to the most efficient cuproptosis induction. Next, the flow cytometry analysis was performed to evaluate the DC-maturation rate in tumor-draining lymph nodes (TDLNs). The DC-maturation rate in ZCH group was 46.2 %, which was higher than CuCl_2_+ZH and CuCl_2_ group, respectively (Fig. 5a and b). Given the important role of the mature DCs in initiating, modulating, and maintaining the immune response, we detected the percentage of helper T cells (CD3^+^CD4^+^ T cells) and cytotoxic T lymphocytes (CD3^+^CD8^+^ T cells) in tumor tissue from mice treated by different nanomaterials. As shown in Fig. 5c–f, the frequency of tumor-infiltrating CD3^+^CD4^+^ and CD3^+^CD8^+^ T cells were 46.2 % and 11.5 % in ZCH group, while they were only 18.5 % and 6.8 % in control group, demonstrating the powerful ability of facilitating cytotoxic T lymphocytes (CTLs) infiltration mediated by ZCH. Meanwhile, the result was further proved by the immunofluorescence staining of sections from tumor tissues further (Fig. 5g and h). Since the cytokines played an important role in anti-tumor immune regulation, we detected the levels of cytokine in serum samples from mice treated by different nanomaterials. Consistent with flow cytometry analysis, the ZCH group showed the highest secretion levels of cytokines (IFN-γ and TNF-α), further illustrating the vigorous cuproptosis-mediated anti-tumor immune responses in vivo (Figs. S21 and S22).

**Fig. 6:**
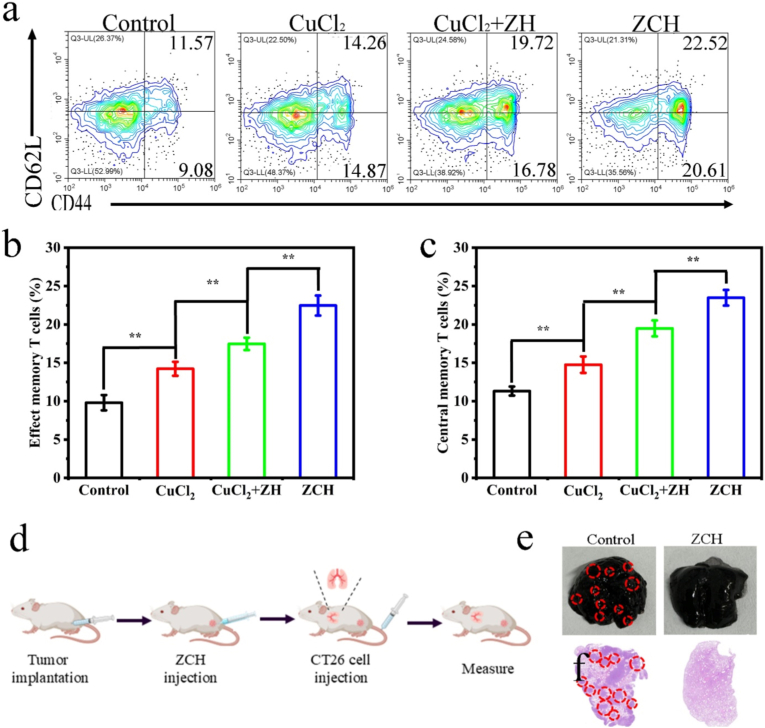
Immunological memory induced by ZCH prevent lung metastasis of CT26 cells. (a) Flow cytometric assay,(b) relative populations of central memory T cells and (c) effector memory T cells in spleen from different groups. (d) Scheme of the therapeutic protocol on the inhibition of lung metastasis. (e) Representative photographs of lung metastasis nodules and (f) H&E-stained images of lung tissues in different treatment groups (Scale bar: 100 μm). ∗p < 0.05, ∗∗p < 0.01, and ∗∗∗p < 0.001.

Immunological memory is one of the hallmarks of adaptive immune responses, which could provide long-term protection to prevent the occurrence of tumor metastasis. Some research revealed that the effective activation of antitumor immunity and induction of tumor ICD could induce tumors to produce in situ tumor vaccines, activating the body's long-term antitumor immune response [[50], [51], [52]]. We detected the effector memory T cells (T_EM_, CD3^+^CD8^+^CD44^+^CD62L^−^) and central memory T cells (T_CM_, CD3^+^CD8^+^CD44^+^CD62L^+^) in spleen from mice treated with different nanomaterials to assess immunological memory mediated by ZCH. Satisfactorily, the percentages of T_EM_ and T_CM_ cells in the ZCH group was 22.5 % and 23.4 %, which were significantly higher than that in control group (9.8 % and 11.3 %), CuCl_2_ group (14.2 % and 14.8 %) and CuCl_2_+ZH group (17.5 % and 19.5 %), respectively, indicating the most efficient long-term memory effect of ZCH (Fig. 6a–c). Considering the fact that the cancer cells transfer into the circulatory system from primary tumor is the main reason of occurrence of tumor metastasis, CT26 bearing-tumor mice were intravenously injected with CT26 tumor cells after finishing the in situ treatment to evaluate ability of ZCH to against lung metastasis (Fig. 6d) [53]. As expected, in contrast to ZCH groups, lots of metastatic nodules were observed in the control groups. Meanwhile, H&E staining of lung tissues further proved this result (Fig. 6e). Overall, these results provide strong evidence that ZCH could activate cuproptosis by accumulation of copper ions, inhibiting glycolysis and breaking intracellular redox homeostasis, further inducing antitumor immune response and immunological memory, and hinder the growth of tumor and lung metastasis.

## 3 Conclusion

In summary, we constructed a novel cuproptosis nano-inducer with the good abilities of copper ions delivery, glycolysis inhibition and breaking intracellular redox homeostasis for triggering efficient cuproptosis-mediated immunotherapy. ZCH could release Cu^2+^ in TME for controllably delivering Cu and generation ROS. Meanwhile, released Zn^2+^ blocked ATP7B by inhibiting glycolysis and production of intracellular ATP, further resulting in the reduction of copper ion efflux. The enhancement of ROS caused by self-supplied H_2_O_2_ of ZCH, breaking the intracellular redox homeostasis and depleting the intracellular GSH for amplifying cuproptosis. Subsequently, the released DAMPs further provoked immune response for achieving highly efficient immunotherapy of colorectal cancer, which created a long-lasting immunological memory, remarkably impeded tumor growth and inhibit lung metastasis. Thus, our research offers a promising nanoplatform for colorectal cancer immunetreatment via ICD mediated by cuproptosis.

## CRediT authorship contribution statement

**Shaopeng Zhang:** Writing – original draft, Visualization, Methodology, Investigation, Conceptualization. **Shaokang Yang:** Investigation. **Mingqi Li:** Investigation. **Hao Zhang:** Investigation. **Yue Cao:** Methodology. **Shiqi Bai:** Methodology. **Wei Li:** Supervision. **Bin Wang:** Methodology. **Donghao Qu:** Methodology. **Ziqian Wang:** Visualization. **Wanying Li:** Investigation. **Yanxu Sun:** Visualization, Supervision. **Daguang Wang:** Writing – review & editing, Supervision, Conceptualization. **Yinghui Wang:** Writing – review & editing, Supervision, Project administration, Conceptualization. **Hongjie Zhang:** Writing – review & editing, Supervision, Conceptualization.

## Declaration of competing interest

The authors declare that they have no known competing financial interests or personal relationships that could have appeared to influence the work reported in this paper.

## Data Availability

Data will be made available on request.
