# Supplementary material for: Copper-enriched zinc peroxides induced cuproptosis through concurrent metabolic and oxidative dysregulation for boosting immunotherapy in colorectal cancer
Source: Mater Today Bio. 2026 Jan 19;37:102830. doi: 10.1016/j.mtbio.2026.102830 (PMC12858360; doi:10.1016/j.mtbio.2026.102830)
Supplement: Multimedia component 1 [file mmc1.docx]

Copper-Enriched Zinc Peroxides Induced Cuproptosis through Concurrent Metabolic and Oxidative Dysregulation for Boosting Immunotherapy in Colorectal Cancer

Shaopeng Zhang^a,b^, Shaokang Yang^a^, Mingqi Li^b^, Hao Zhang^c^, Yue Cao^d^, Shiqi Bai^e^, Wei Li^a^, Bin Wang^d^, Donghao Qu^d^, Ziqian Wang^d^, Wanying Li^f^, Yanxu Sun^g^, Daguang Wang^a^*, Yinghui Wang^f^*, Hongjie Zhang^f^*.

a. Department of Gastrocolorectal Surgery, General Surgery Center, The First Hospital of Jilin University, Changchun 130021, PR China.

b. Department of Colorectal Surgery, Harbin Medical University Cancer Hospital, No.150 Haping Road, Harbin, Heilongjiang, 150081, China.

c. Department of Central Laboratory, The Affiliated Huaian No.1 People's Hospital Nanjing Medical, Huai'an, 223300, Jiangsu Province, China.

d. Department of Neurosurgery, The First Hospital of Jilin University, Changchun 130021, PR China.

e. Genetic Diagnosis Center, The First Hospital of Jilin University, Changchun 130021, PR China.

f. State Key Laboratory of Rare Earth Resource Utilization, Changchun Institute of Applied Chemistry (CIAC), Chinese Academy of Sciences, Changchun 130022, China.

g. Operating room Surgery, Harbin Medical University Cancer Hospital, No.150 Haping Road, Harbin, Heilongjiang, 150081, China.

* Email: yhwang@ciac.ac.cn; dgwang@jlu.edu.cn; hongjie@ciac.ac.cn

**1.Experimental Section**

**1.1 Chemicals and Reagents**

Zinc acetate (Zn (CH_3_COO)_2_⋅2H_2_O) , Tetrathiomolybdate (TTM) and Copper chloride (CuCl_2_·2H_2_O) were obtained from Aladdin Reagent Co, Ltd (Shanghai, China). Terephthalic acid (TA) was obtained from Macklin Biotechnology Co, Ltd (Shanghai, China). Glutathione Peroxidase Assay Kit, calcein acetoxymethyl ester (calcein-AM), ATP assay kit, Red Blood Cell Lysis Buffer, Cell lysis buffer for Western and IP, propidium iodide (PI) and DAPI were obtained from Beyotime Biotechnology Co, Ltd (Shanghai, China). Mouse IL-4 and mouse GM-CSF were obtained from Beijing Solarbio Science & Technology Co., Ltd. (Beijing, China). The ROSGreenTM H_2_O_2_Probe, MitoSOX Red Mitochondrial Superoxide Indicator and Zinquin ethyl ester were obtained from Maokang Biotechnology Co.Ltd (Shanghai, China). Cell counting kit-8 (CCK-8) and fluorescein isothiocyanate isomer (FITC-I) were purchased from Beijing Bioss Biotechnology Co., Ltd. (Beijing, China). Hyaluronic acid (HA) was obtained from TCI Chemical Industry Development Co, Ltd(Shanghai, China). GSH Assay Kit and Lactate (LA) Assay Kit was purchased from Nanjing Jiancheng Bioengineering Institute (Nanjing, China). Dimethylformamide (DMF), Hydrogen peroxide (H_2_O_2_), ethanol was provided by Sinopharm Chemical Reagent Co. Ltd (Shanghai, China). Anti-DLAT antibody, anti-LIAS antibody, anti-ATP7B antibody and anti-FDX1 antibody, anti-GAPDH antibody and anti-LDHA antibody were provided by Servicebio Co. Ltd (Beijing, China). Mouse HMGB-1(High-mobility group protein B1) ELISA Kit was purchased from Elabscience Biotechnology Co. Ltd (Wuhan,China). Mouse IFN-γ Precoated ELISA Kit and Mouse TNF-α Precoated ELISA Kit were purchased from Dakewe Biotech Co. Ltd (Shenzhen,China).All the antibody for flow cytometry assay were from BioLegend.

**1.2 Cell lines and animals**

CT26 colorectal cancer cells and L929 cells were cultured with cell culture medium containing 10% FBS and 100 units per mL of penicillin and streptomycin at 37 °C in a 5% CO_2_ and 95% humidity atmosphere. The female BALB/c mice were obtained from Changsheng Biotechnology. The animal experimental protocol was reviewed and approved by Experimental Animal Ethics of The First Hospital of Jilin University.

**1.3 Preparation of ZnO_2_-Cu@HA Nanoparticle**

To produce the ZnO_2_, 0.46 g of zinc acetate was dissolved in 49 mL of deionized water. Then, 2 mL of H_2_O_2_ (30%) was added to zinc acetate solution. Then, the mixture was suddenly introduced into a superheated plate at a temperature of 280℃. Finally, the ZnO_2_ precipitate was obtained by centrifugation at 12000 rpm for 5 minutes. The products were washed several times with deionized water.

For the synthesis of ZnO_2_-Cu, 2 mL anhydrous ethanol solution of ZnO_2_ (5.0mg/mL) was dissolved into the distilled water (7.4 mL) followed sonicated for 10 min. Then, 600 μL anhydrous ethanol solution of CuCl_2_·2H_2_O(100 mM) was added to the mixture. After reacting for 60 min at room temperature under violent stirring, ZnO_2_-Cu was obtained by centrifugation and washed with deionized water (12,000 rpm, 5 min × 3).

For the synthesis of ZnO_2_-Cu@HA (ZCH), 20 mg of ZnO_2_-Cu was added to anhydrous ethanol (8 mL), then adding 2 mL deionized water solution of HA (2.5 mg/mL) to stir for 24 h. ZCH was obtained and purified with deionized water for several cycles.

For the synthesis of ZnO_2_@HA (ZH), 20 mg of ZnO_2_ was added to anhydrous ethanol (8 mL), then adding 2 mL deionized water solution of HA (2.5 mg/mL) to stir for 24 h. ZH was obtained and purified with deionized water for several cycles.

**1.4 Characterization**

Transmission electron microscopic (TEM) images were obtained from a TECNAI G2 high resolution transmission electron microscope. Powder X-ray diffraction (XRD) was tested by a Bruker D8 Focus Powder X-ray diffraction. Inductively coupled plasma -optical emission spectroscopy (ICP-OES) were performed to test the contents of element. Based on Bruker Vector 22 spectrometer, Fourier transform infrared (FTIR) spectra were performed. Fluorescence spectrometer (F-7000) was applied to detect •OH. The zeta potential was measured by a Zetasizer nano series (Malvern). X-ray photoelectron spectroscopy (XPS) spectra were performed on an ESCALAB-MKII 250 photoelectron spectrometer.

**1.5 pH responsive degradation of ZCH**

To evaluate the pH responsive degradation of ZCH, ZCH was dispersed in solution with different pH values (7.4 and 6.5). After 24 h, ZCH was taken out and dropped on the carbon coated copper grids for TEM analysis

**1.6 The release of copper and zinc from ZCH**

The release profiles of copper and zinc were studied in solution with different pH values (7.4 and 6.5) within thermostatic shaker (200 rpm/min, 37℃). At 1 h, 2 h, 4 h, 8 h, 16 h and 24 h, 1 mL of solution was extracted and centrifugated (12,000 rpm, 5 min) for further testing the content of copper and zinc by ICP-OES.

**1.7 Detection of extracellular H_2_O_2_ generation**

ROSGreenTM H_2_O_2_ probe was performed to assess the release of H_2_O_2_. 200 μg/mL ZCH was incubated in PBS (pH 7.4 and pH 6.5) for 12 h at room temperature. After centrifugation supernatant was taken and then 5 μM ROSGreenTM H_2_O_2_ probe was added. The emission spectra were recorded on Fluorescence Spectrophotometer (F7000) using excitation wavelength of 490 nm.

**1.8 Detection of extracellular •OH generation**

Different concentrations of ZCH (0, 10 ppm, 20 ppm, 40 ppm, 80 ppm and 160 ppm) were added to PBS (pH=6.5) containing terephthalic acid (TA) (22 ppm) and GSH (2 mM). After 12 h, the fluorescence intensity at 435nm of supernatant was measured by a fluorometer.

**1.9 Detection of GSH-depletion**

ZCH (0, 20 ppm, 40 ppm, 60 ppm and 80 ppm) were added to PBS (pH=6.5) containing GSH (1 mM) for 60 min. The supernatant was obtained via centrifugation for the next measurement of absorbance at 412 nm by a UV-vis spectrophotometer by GSH Assay Kit.

**1.10 Cell uptake**

A fluorescence microscope was used to assess the cellular uptake behavior. Briefly, CT26 cells were implanted into a 96-well plate for 24 h. Then, the CT26 cells were treated with ZCH (concentrations of Cu in nanoparticles: 5 ppm) for different time (1,2,4, and 8h), followed by being washed with PBS, before eventual DAPI staining for 15min. Finally, fluorescence microscopic images were obtained by a fluorescence microscope.

**1.11 Cell biocompatibility in vitro**

The biocompatibility of ZCH was evaluated in mouse fibroblast cells (L929 cells). L929 cells were implanted into a 96-well plate for 24 h. Then, the L929 cells were incubated with different concentrations of Cu in ZCH (0, 1 ppm, 2 ppm, 3 ppm, 4 ppm, 5 ppm) for 24 h. Finally, the CCK-8 assay was applied to evaluate cell viability.

**1.12 Cell cytotoxicity in vitro**

CT26 cells were implanted into a 96-well plate and cultivated for 24h. Subsequently, the CT26 cells were divided four groups (Control, CuCl_2,_ CuCl_2_+ZH, ZCH and ZCH+TTM), and treated with different concentrations of Zn and Cu in nanoparticles (Zn: 0 ppm, 2.2 ppm, 4.4 ppm, 6.6 ppm, 8.8 ppm and 11 ppm; Cu: 0 ppm, 1 ppm, 2 ppm, 3 ppm, 4 ppm and 5 ppm) for 24h. Finally, the CCK-8 assay was employed to evaluate the viability of cells.

**1.13 Live-dead Cell Staining.**

CT26 cells were implanted into a 96-well plate and cultivated for 24h, followed by treated with 1640 culture medium, CuCl_2,_ CuCl_2_+ZH, ZCH and ZCH+TTM (concentrations of Zn: 11 ppm, concentrations of Cu: 5 ppm) for 24 h. Subsequently, CT26 cells were stained with calcein-AM and PI, and washed with PBS for the final monitoring by fluorescence microscopy.

**1.14 Migration assay**

A Transwell system was used for the migration assay. Specifically, CT26 cells were implanted into transwell donor chamber for 4 h, and treated with 1640 culture medium, CuCl_2,_ CuCl_2_+ZH, ZCH and ZCH+TTM (concentrations of Zn: 6.6 ppm, concentrations of Cu: 3 ppm) for 24 h. After gently swabbing the interior of the donor chamber with a cotton swab, the cells were fixed with 4% paraformaldehyde solution and subsequently stained with 0.1% crystal violet solution. The cell migration were then observed by fluorescent microscope.

**1.15 Detection of intracellular Zn^2+^**

Zinquin ethyl ester was employed to evaluate the release of Zn^2+^ in cell. Specifically, CT26 cells were implanted into 96-well cell plates for 24h, and treated with 1640 culture medium, CuCl_2,_ CuCl_2_+ZH and ZCH (concentrations of Zn: 6.6 ppm, concentrations of Cu: 3 ppm) for 12 h. Then, CT26 cells were stained with Zinquin ethyl ester further imaged by fluorescent microscope.

**1.16 Detection of intracellular LA, GSH and ATP**

CT26 cells were implanted into 96-well cell plates and cultivated for 24 h, and treated with 1640 culture medium, CuCl_2,_ CuCl_2_+ZH and ZCH (concentrations of Zn: 6.6 ppm, concentrations of Cu: 3 ppm) for 12 h. Then, Then, the cells were collected and broken by Cell lysis buffer for Western and IP for next measure. The GSH Assay Kit, ATP Assay Kit and LA Assay Kit were used to assess intracellular GSH content, ATP and LA content, respectively. Meanwhile, the supernatants were also collected to evaluate the extracellular LA and ATP.

**1.17 Detection of intracellular Cu content**

CT26 cells were implanted into 96-well cell plates and cultivated for 24 h, and treated with 1640 culture medium, CuCl_2,_ CuCl_2_+ZH and ZCH (concentrations of Zn: 6.6 ppm, concentrations of Cu: 3 ppm) for 12 h. Then, Then, the cells were collected and added to aqua regia for ICP-OES.

**1.18 Detection of intracellular ROS**

CT26 cells were implanted into 96-well cell plates and cultivated for 24 h, and treated with 1640 culture medium, CuCl_2,_ CuCl_2_+ZH and ZCH (concentrations of Zn: 6.6 ppm, concentrations of Cu: 3 ppm) for 12 h. Then, CT26 cells were stained by DCFH-DA for 25 min, and washed with PBS, further imaged by fluorescent microscope.

**1.19 Detection of intracellular mitochondrial ROS (mitoROS)**

CT26 cells were implanted into 96-well cell plates and cultivated for 24 h, and treated with 1640 culture medium, CuCl_2,_ CuCl_2_+ZH and ZCH (concentrations of Zn: 6.6 ppm, concentrations of Cu: 3 ppm) for 12 h. Afterwards, the mitoSOX fluorescence probe was employed to determine the intracellular mitochondrial ROS (mitoROS) level.

**1.20 Detection of DLAT aggregation in vitro**

CT26 cells were implanted into 96-well cell plates and cultivated for 24 h, and treated with 1640 culture medium, CuCl_2,_ CuCl_2_+ZH and ZCH (concentrations of Zn: 6.6 ppm, concentrations of Cu: 3 ppm) for 12 h. After that, the cells were sequentially fixed with 4% paraformaldehyde, incubated with the DLAT antibody at 4 ℃ overnight, treated with secondary antibody at room temperature for 1 h, stained with DAPI for 15 min, and imaged by CLSM.

**1.21 Detection of CRT expression.**

CT26 cells were implanted into 96-well cell plates and cultivated for 24 h, and treated with 1640 culture medium, CuCl_2,_ CuCl_2_+ZH and ZCH (concentrations of Zn: 6.6 ppm, concentrations of Cu: 3 ppm) for 12 h. The treated CT26 cells were sequentially fixed with 4% paraformaldehyde, incubated with anti-CRT antibody (Bioss) at 4 ℃ overnight, treated with secondary antibody at room temperature for 1 h, stained with DAPI for 15 min, and imaged by CLSM.

**1.22 Detection of HMGB-1**

CT26 cells were implanted into 96-well cell plates and cultivated for 24 h, and treated with 1640 culture medium, CuCl_2,_ CuCl_2_+ZH and ZCH (concentrations of Zn: 6.6 ppm, concentrations of Cu: 3 ppm) for 12 h. The release of HMGB-1 was detected by the corresponding Assay Kit according to the manufacture’s protocols.

**1.23 Detection of DCs maturation in vitro**

Firstly, Bone marrow-derived dendritic cells (BMDCs) were obtained by flushing the femur and tibia with PBS. Then, the BMDCs was cultivated by 1640 culture medium including the mouse GM-CSF and mouse IL-4 for 10 days. By a Transwell system, residues of CT26 cells treated with 1640 culture medium, CuCl_2,_ CuCl_2_+ZH and ZCH (concentrations of Zn: 6.6 ppm, concentrations of Cu: 3 ppm) medium solution were incubated with DCs. Then, DCs were stained with APC anti-mouse CD11c, PE anti-mouse CD80, and FITC anti-mouse CD86 for the next flow cytometry analysis (BD Canto II).

**1.24 Analysis of expression of intracellular protein by Western blotting assay**

The expression of ATP7B, FDX1, GAPDH, LDHA, LIAS in CT26 cells was evaluated by western blot. CT26 cells were implanted into 6-well cell plates, and treated with 1640 culture medium, CuCl_2,_ CuCl_2_+ZH and ZCH (concentrations of Zn: 6.6 ppm, concentrations of Cu: 3 ppm) for 12h. Then, the trypsin was employed to digest cells for the subsequent protein extraction. BCA Protein Assay Kit was employed to measure protein concentration. Subsequently, protein from each sample were separated by SDS-PAGE and transferred to the PVDF membranes for subsequent incubating with corresponding primary antibodies and HRP-labeled second antibodies. Finally, the results were acquired by ECL chemiluminescence kit.

**1.25 Biological toxicity in vivo**

The mice were intravenously injected with ZCH (100 μL, concentrations of Cu: 100 ppm). The whole blood was collected from the orbital venous plexus on 30-day post-injection for the subsequent blood routine and biochemistry examination.

**1.26 In vivo** **antitumor efficacy**

The female BALB/c mice were employed to construct tumor model, and were randomly divided into 4 groups (n=3) for different treatment: (a) PBS solution; (b) CuCl_2_ (266 ppm); (c) CuCl_2_ (266 ppm) + ZH (325 ppm); (d) ZCH (588 ppm); (100 μL, concentrations of Cu: 100 ppm, concentrations of Zn: 220 ppm). During the treatment, body weight and tumor volume were measured every other day. After 15-day treatment, tumors and organs including heart, liver, spleen, kidney and lung were further studied by H&E staining. Meanwhile, the tumors were collected for immunofluorescence assay to detect the expression of DLAT, CD4, CD8, CRT and HMGB1 in tumor.

**1.27 Flow cytometric analysis and cytokine detection**

To systematically investigate the antitumor immune responses, the lymph nodes, spleen and tumor were collected for flow cytometric analysis. The cells were stained with APC anti-mouse CD3, FITC anti-mouse CD8a, and PE anti-mouse CD4 antibodies to differentiate cytotoxic T lymphocytes (CTLs, CD3^+^CD4^−^CD8^+)^ and helper T cells (CD3^+^CD4^+^CD8^−^). The cells were stained with APC anti-mouse CD11c, PE anti-mouse CD80, and FITC anti-mouse CD86 to differentiate the mature DCs (CD11c^+^CD80^+^CD86^+^). The cells were stained with APC anti-mouse CD3, FITC anti-mouse CD8a, PE anti-mouse CD44 and PerCP-Cy5.5 anti-mouse CD62L to identify central memory T cells (T_CM_, CD3^+^CD8^+^ CD44^+^CD62L ^+^) and effector memory T cells (T_EM_, CD3^+^CD8^+^ CD44^+^CD62L^-^). The stained cells were detected by flow cytometry analysis (BD Canto II). The levels of cytokines (IFN-γ and TNF-α) in serum were measured by with the corresponding ELISA Kit .

**1.28 Lung** **metastasis studies**

The untreated BALB/c mice and ZCH cured mice were intravenously injected CT26 cells. After 30 d, lungs were stained by India ink for metastatic nodule counting. In addition, lungs sections were harvested from each group and further studied by H&E staining assay.

**1.29 Statistical analysis**

Experimental data are presented as means ± SD. Comparison analysis between groups was calculated by t-test with SPSS. *P<0.05, **P < 0.01 and ***P < 0.001 are considered statistically significant.


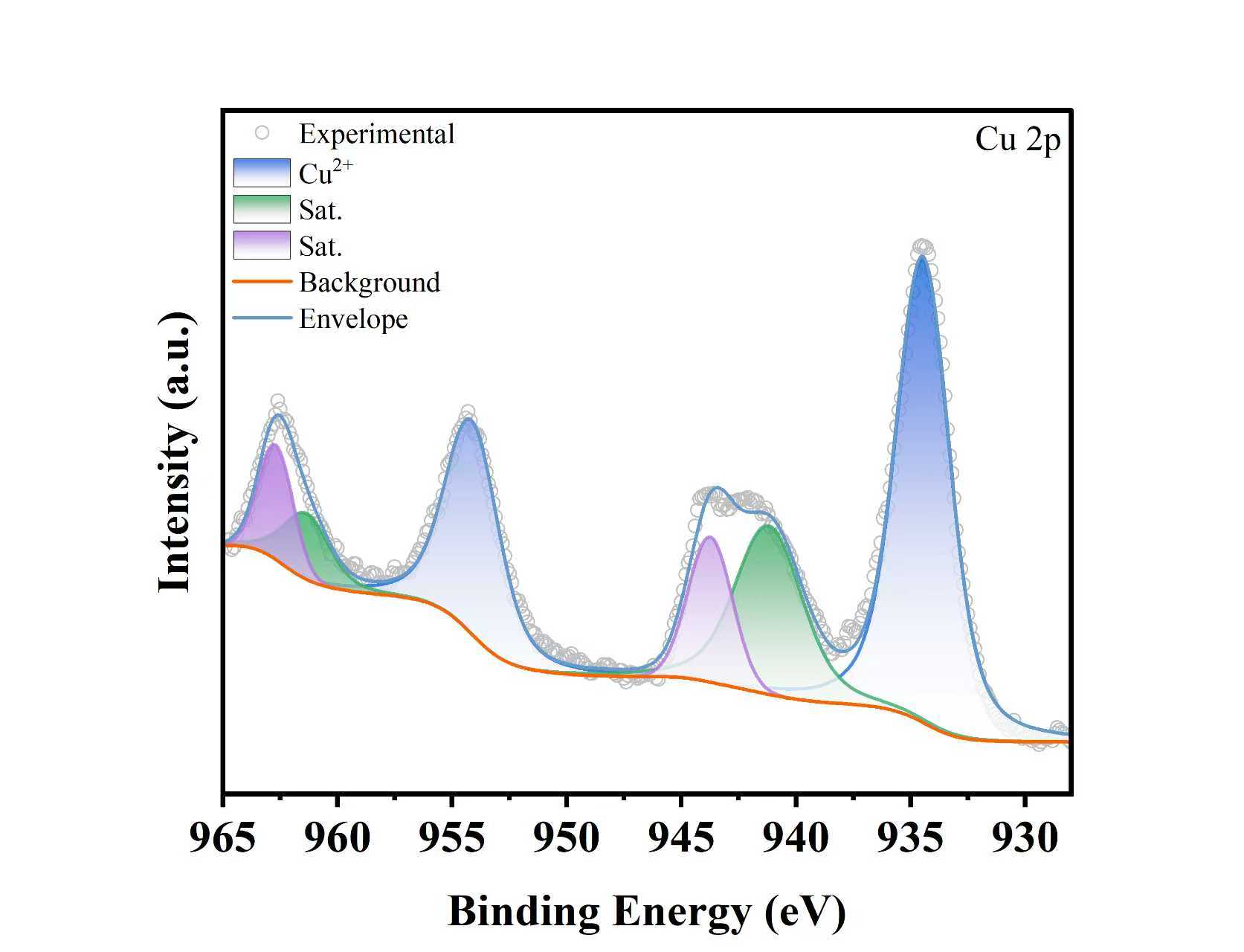


**Figure S1.** Cu 2p spectrum of ZnO_2_-Cu NPs





**Figure S2.** Zn 2p spectrum of ZnO_2_-Cu NPs





**Figure S3.** Hydrodynamic size of ZCH in H_2_O, PBS and RPMI 1640 medium.





**Figure S4.** Hydrodynamic size of ZCH being incubated in H_2_O, PBS or RPMI 1640 medium for 7 days.


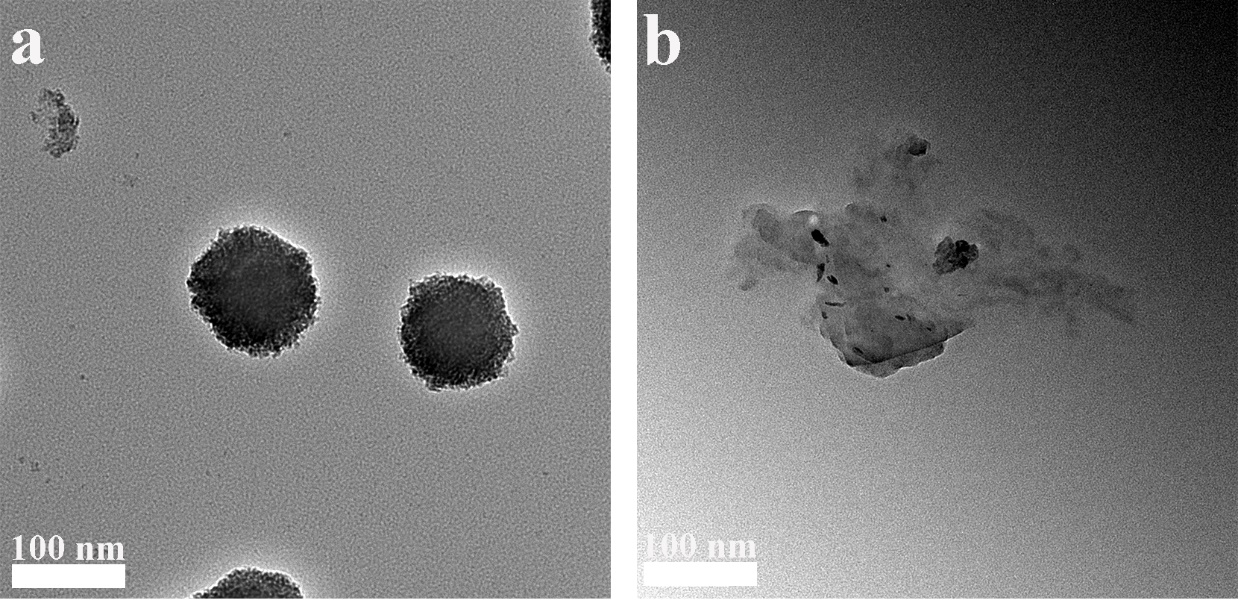


**Figure S5.** TEM images of degraded ZCH NPs at different condition.(a)pH=7.4. (b) pH=6.5.





**Figure S6.** The release behavior of Cu element from ZCH NPs under different conditions.





**Figure S7.** The mean fluorescence intensity of FITC


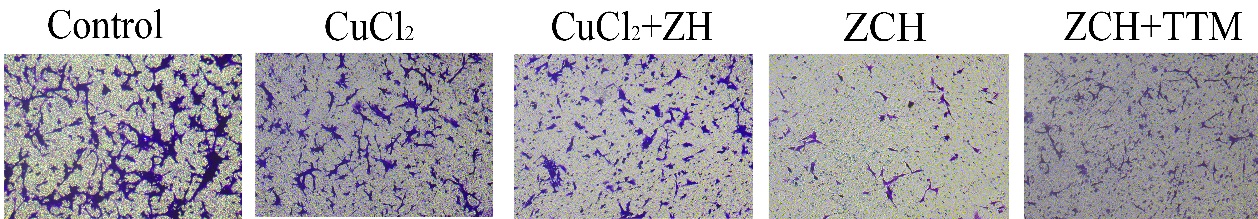


**Figure S8.** The result of migration assay for different nanomaterials





**Figure S9.** The mean fluorescence intensity of Zinquin ethyl ester.





**Figure S10.** The detection of extracellular LA content of CT26 cells in different groups.


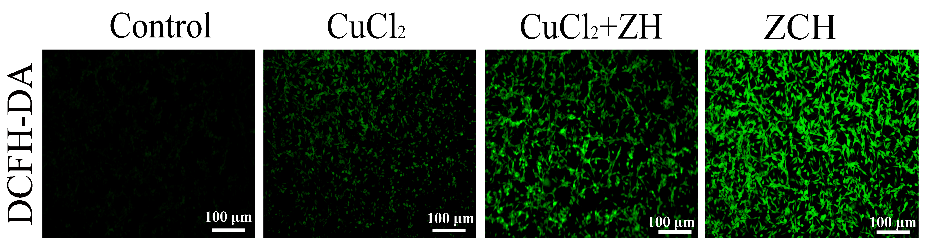


**Figure S11.** The detection of ROS in CT26 cells after treated with different nanomaterials. (Scale bar: 100 µm)





**Figure S12.** The mean fluorescence intensity of DCFH-DA.





**Figure S13.** The mean fluorescence intensity of MitoSOX.





**Figure S14.** The mean fluorescence intensity of DLAT.





**Figure S15.** The mean fluorescence intensity of CRT.


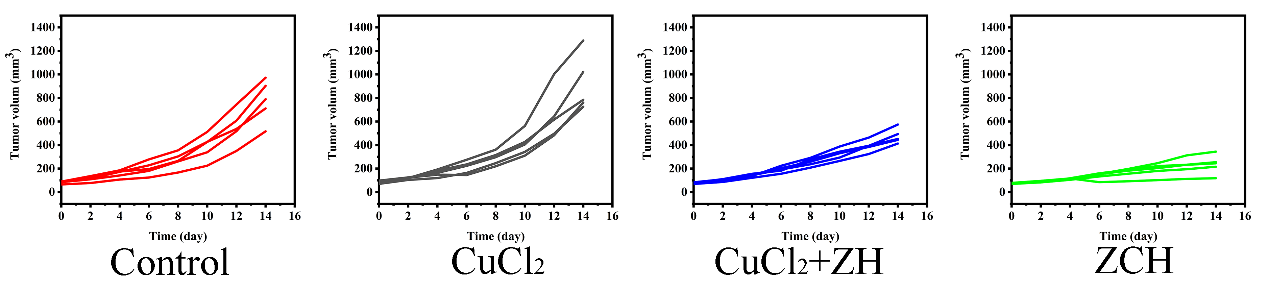


**Figure S16.** Individual tumor growth curves after different treatments.


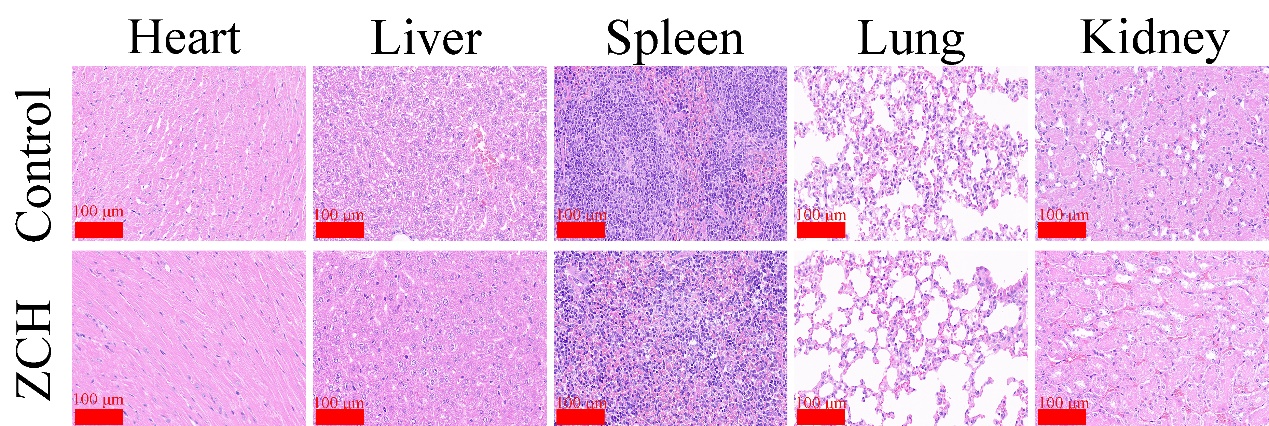


**Figure S17.** H&E staining images of major organs from mice in ZCH and control group (Scale bar: 100 μm).


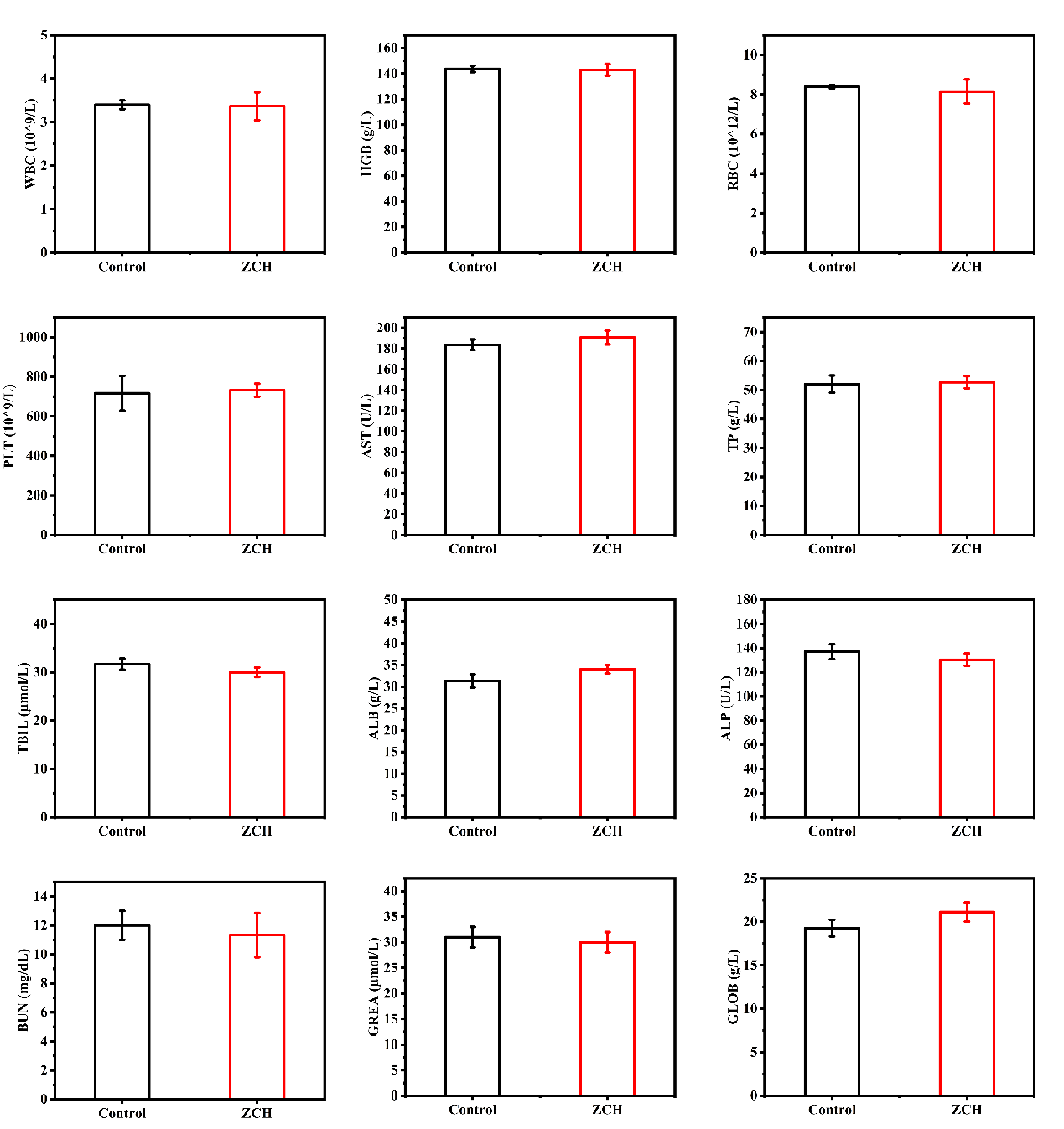
**Figure S18.** Routine blood analysis after intravenous injection of ZCH and PBS at 30 d.


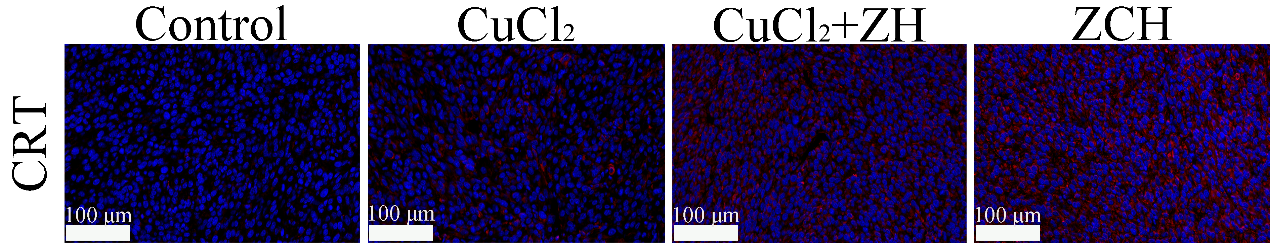


**Figure S19.** The CRT staining images of tumors from mice in various groups (Scale bar: 100 μm).


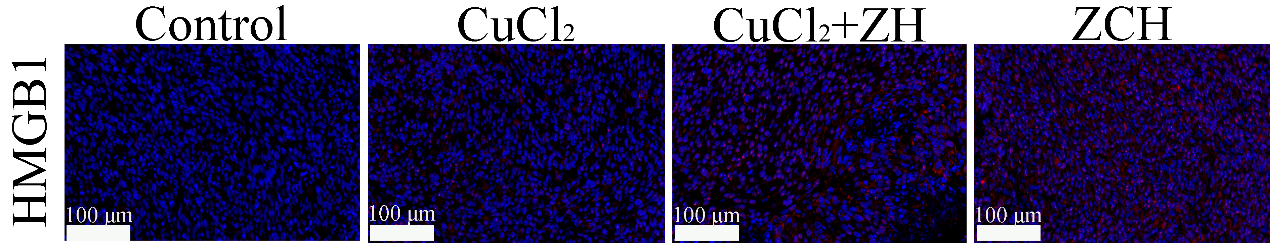


**Figure S20.** The HMGB1 staining images of tumors from mice in various groups (Scale bar: 100 μm).





**Figure S21.** The levels of TNF-α in serum samples after mice treated with with different nanomaterials.





**Figure S22.** The levels of IFN-γ in serum samples after mice treated with with different nanomaterials.

**Table. S1 Main elements contents of ZH and ZCH (1000 μg/mL) measured by ICP-OES.**

|  | **Zn** | **Cu** |
| --- | --- | --- |
| **ZH** | **675.2±8.9 μg/mL** | **0.0±0.0 μg/mL** |
| **ZCH** | **366.3±11.4 μg/mL** | **170.7±8.5 μg/mL** |
